# The role of Gadd45b in neurologic and neuropsychiatric disorders: An overview

**DOI:** 10.3389/fnmol.2022.1021207

**Published:** 2022-10-13

**Authors:** Xiao-yue Shen, Shu-han Shi, Heng Li, Cong-cong Wang, Yao Zhang, Hui Yu, Yan-bin Li, Bin Liu

**Affiliations:** ^1^Department of Neurology, The First Affiliated Hospital of Shandong First Medical University and Shandong Provincial Qianfoshan Hospital, Jinan, China; ^2^The First Clinical Medical College, Shandong University of Traditional Chinese Medicine, Jinan, China; ^3^College of Traditional Chinese Medicine, Shandong University of Traditional Chinese Medicine, Jinan, China; ^4^School of Clinical Medicine, Weifang Medical University, Weifang, China

**Keywords:** neuropsychiatric disorders, cerebral ischemia, mental illness, neurologic disorders, BDNF, Gadd45b

## Abstract

Growth arrest and DNA damage-inducible beta (Gadd45b) is directly intertwined with stress-induced DNA repair, cell cycle arrest, survival, and apoptosis. Previous research on Gadd45b has focused chiefly on non-neuronal cells. Gadd45b is extensively expressed in the nervous system and plays a critical role in epigenetic DNA demethylation, neuroplasticity, and neuroprotection, according to accumulating evidence. This article provided an overview of the preclinical and clinical effects of Gadd45b, as well as its hypothesized mechanisms of action, focusing on major psychosis, depression, autism, stroke, seizure, dementia, Parkinson’s disease, and autoimmune diseases of the nervous system.

## Introduction

**FIGURE 1:**
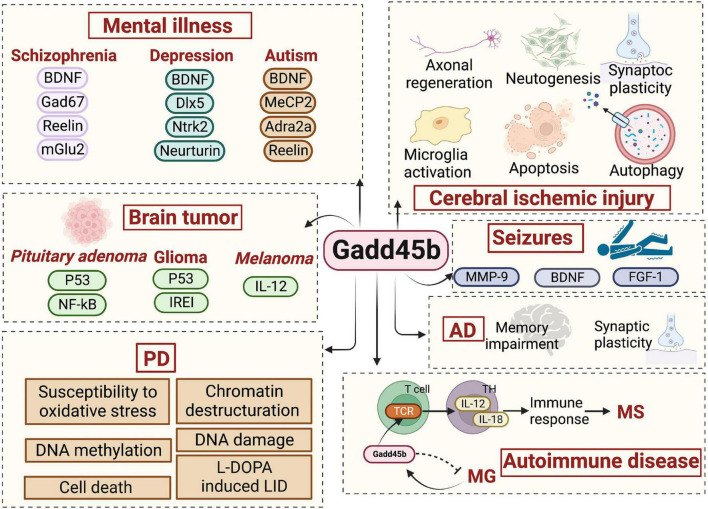
The role of growth arrest and DNA damage-inducible beta (Gadd45b) in neurologic and neuropsychiatric disorders.

The growth arrest DNA damage-inducible gene (Gadd45) family consists of Gadd45a, Gadd45b, and Gadd45g. All of them are nuclear proteins with a molecular weight of 18 KD. These molecules are widely expressed in the nervous system. And it is required for the stress response, apoptosis, and mitosis of neuronal and glial cells (Kearsey et al., 1995; Hildesheim et al., 2002). The Gadd45 protein is mainly involved in regulating the cell cycle, cell apoptosis, proliferation, and DNA damage repair and may be interested in DNA demethylation (Barreto et al., 2007; Liebermann and Hoffman, 2007; Ma et al., 2009). DNA methylation is a major epigenetic factor, the specific temporal control of which is critical for the growth and differentiation of the mammalian central nervous system (CNS) (Fan et al., 2001, 2005; Feng et al., 2005; Golshani et al., 2005). DNA methylation is a crucial regulatory process involved in brain formation, learning, memory, and disorder (Rizzardi et al., 2019), and it is known to be modified in individuals with neuropsychiatric disorders such as schizophrenia, addiction, and Alzheimer’s disease (Jeong et al., 2021). Writers, erasers, and readers are the three main types of enzymes that create, identify, and remove DNA methylation. DNA methylation is the mechanism by which methyl groups can be covalently attached to the 50 positions on cytosine nucleotides, resulting in the formation of 5-methylcytosine (5mC). Demethylation remains incompletely understood but includes DNA damage repair enzymes and various intermediates, such as 5-hydroxymethylcytosine (5-hmC) (Tahiliani et al., 2009). Specifically, the conversion of 5mC to 5hmC may be mediated by base-excision and repair (BER) or after the transformation of 5hmC to 5-hydroxymethyluracil (5hmU) by the activation-induced deaminase (AID). As a member of the Gadd45 family, Gadd45b, is a demethylase, participating in the BER of mutated cytosines and may perform a similar function in neurons. Besides, Gadd45b has been proven to have a crucial function in the nervous system and immunity, epigenetic DNA demethylation, neuroplasticity, and neuroprotection. Conclusively, Gadd45b is commonly implicated in neurologic and neuropsychiatric diseases because of its critical involvement in brain plasticity (Figure 1).

Neurological diseases are a group of disorders or abnormalities in the nervous system, commonly including stroke, seizure, dementia, Parkinson’s disease and multiple sclerosis. Meanwhile, brain diseases often result in psychological symptoms, including schizophrenia, depression, autism, etc. Neuropsychiatric diseases mainly affect cognitive, emotional, and behavioral disorders. Although neurological and neuropsychiatric illnesses are two distinct disease categories, their etiology and pathophysiology often overlap Gadd45b-dependent molecular signaling pathways.

## The role of Gadd45b in neuropsychiatric disorders

### Major psychosis

Major psychosis, which refers chiefly to schizophrenia and bipolar disorder, is a severe, recurrent, and chronic mental condition. These disorders are not only a cause of morbidity but also increased mortality, which results from a relatively high rate of suicide and a shorter life span, due mainly to the medical complications of these illnesses.

**FIGURE 2:**
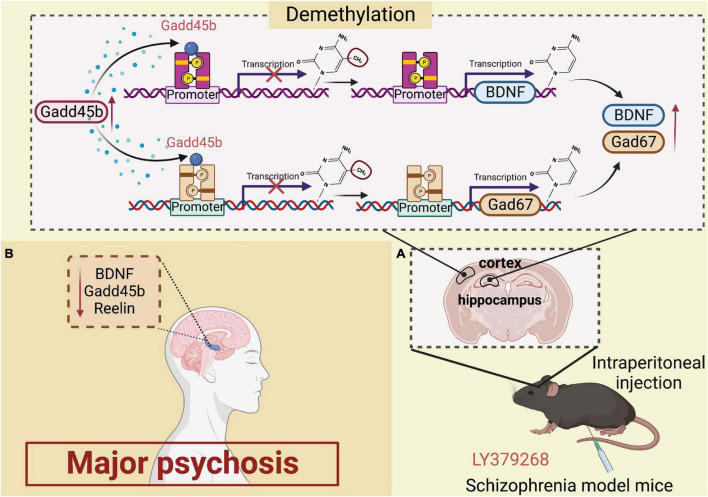
Roles of growth arrest and DNA damage-inducible beta (Gadd45b) in major psychosis. **(A)** Increased expression of gadd45b in the hippocampal region is detected in schizophrenic mice after intraperitoneal injection of ly379268, which alters the Demethylation of BDNF and Gad67, increasing BDNF and Gad67 activation in large amounts. **(B)** Decreased Gadd45b, BDNF and Reelin expression in the brain of patients with major psychosis (Figure was created with BioRender.com).

Multiple investigations over the last decade have identified Gadd45b in the pathogenesis of psychosis of the major psychosis. Patients with psychiatric disorders were shown to have increased Gadd45b-stained cells in prefrontal cortex layers II, III, and V. This research demonstrated that individuals with psychiatric disorders bind less Gadd45b to the BDNF promoter (Gavin et al., 2012). In addition, the scientists showed a decrease in BDNF mRNA expression and an increase in 5-methylcytosine and 5-hydroxymethylcytosine at its promoter. Similarly, the Gadd45 protein is also engaged in regulating histone modifications in the pathological process and therapy of schizophrenia (Guidotti et al., 2009; Labrie et al., 2012). Altered metabotropic glutamate receptor (mGlu) activity is followed by DNA methylation dynamics, as activation of subclass II (composed of mGlu2 and mGlu3 types) attenuates presynaptic glutamate activity, which is linked with antipsychotic effects, according to prior studies (Conn et al., 2009; Kurita et al., 2012). In the mouse frontal cortex and hippocampus, intraperitoneal injection of LY379268 (a brain-permeable mGlu2/3 activator) increases the expression of the Gadd45b mRNA and restores social function. LY379268 also enhances the specific binding of Gadd45b to BDNF and glutamate decarboxylase 67 (GAD67) promoter regions (Matrisciano et al., 2011). Thus, evidence suggests Gadd45b as a viable target for treating schizophrenia. Additionally, Gadd45b is sometimes considered DNA demethylase. Gadd45b binds differentially to the BDNF and GAD67 promoters, altering the demethylation of the BDNF and GAD67 promoters. Notably, Gadd45b levels are increased in mice administered antipsychotic drugs (Guidotti et al., 2011; Matrisciano et al., 2011). Gadd45b appears to control methylation and upregulate reelin, GAD67, and BDNF expression in the brains of individuals with psychosis. These findings imply that Gadd45b is involved in altering brain circuits in patients with major psychosis and modulates antipsychotic treatment (Figure 2).

### Chronic stress and depression

Depression is a prevalent mental illness. According to estimates, 5% of individuals worldwide experience depression (Evans-Lacko et al., 2018). Nevertheless, mechanisms involved in depression pathophysiology are not yet fully understood, and current therapies are inadequate for a substantial proportion of patients with depression (Evans-Lacko et al., 2018; Hasin et al., 2018). Depression severely affects the incidence and prognosis of many common general medical comorbidities, such as stroke. Nevertheless, mechanisms involved in depression pathophysiology are not yet fully understood, and current therapies are inadequate for a substantial proportion of patients with depression. Multiple mechanisms contribute to the pathophysiology and etiology of depression, such as microglia activation, neuroinflammation, and reduced neurogenesis in the hippocampus (Ménard et al., 2016).

Restricted hippocampus neurogenesis elicits depressive-like effects (Kraus et al., 2017). Neurogenesis is governed by substances like BDNF, which is absent in patients with major depression. Anti-depressant treatments, whether pharmacological or psychosocial, restore BDNF levels in individuals with depression (Molendijk et al., 2014; Gururajan et al., 2016). In particular, Gadd45b contributes to BDNF promoter demethylation by enabling the elimination of 5HMC (Gavin et al., 2015).

Likewise, in the Flinders Sensitive Line (FSL) genetic rodent model of depression, an observed trend of decreased Gadd45b mRNA levels in these hypermethylated untreated rats is consistent with the demethylating function of Gadd45b in the adult brain (Melas et al., 2012). In unpredictable chronic mild stress (UCMS) mice, a model for depression, the expression of Gadd45b was down-regulated in the hippocampus by the growth factor β (TGF-β) signaling pathway. This process was involved in the transcriptional regulation of genes (Grassi et al., 2017).

Furtherly, Yin et al. identified a substantial correlation between Gadd45b knockdown and depressive-like behaviors following ischemic stroke. They determined that Gadd45b could play a role in neuronal remodeling activity-related neurogenesis in the hippocampus of adult rats, alleviating poststroke depression through the BDNF signaling pathway (Yin et al., 2021). These findings suggest that Gadd45b is involved in antidepressant-like activity induction.

Electroconvulsive therapy (ECT) was claimed to be an effective treatment for depression (Rasmussen, 2011; Azis et al., 2019). Likewise, ECT has been discovered to trigger the gene Gadd45b, which plays a part in BDNF demethylation in the dentate gyrus of the hippocampus formation (Ma et al., 2009). After ECT treatment, an analysis of the gene expression profiles of hippocampal dentate gyrus granulosa cells revealed that BDNF, neuropeptide Y, and Gadd45b were up-regulated (Ploski et al., 2006). Accordingly, Gadd45b, generated by ECT, at least in part mediates the therapeutic benefits of ECT on depression.

On the contrary, a recent study showed the pro-depressant role of Gadd45b on social behaviors through chronic social defeat stress (Labonté et al., 2019). Gadd45b intervenes in depression by modifying the DNA methylation levels of GAD67, recombinant neurotrophic tyrosine kinase receptor type 2 (Ntrk2), neurturin, and distal-less homeobox 5 (Dlx5). Gadd45b expression was considerably elevated in the nucleus accumbens of susceptible mice in response to persistent social defeat stress. Also, the over-activation of Gadd45b promoted depressed behavior in mice. Moreover, this study revealed that Gadd45b is a novel downstream molecular target of BDNF in the nucleus accumbens.

**FIGURE 3:**
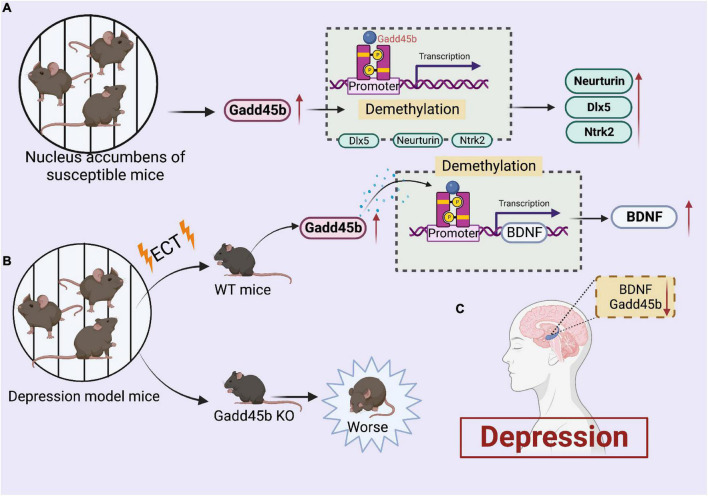
Roles of growth arrest and DNA damage-inducible beta (Gadd45b) in depression. **(A)** Increased gadd45b expression in the brain of nucleus accumbens of susceptible mice alters the demethylation of Neurturin, Dlx5, and Ntrk2, resulting in increased Neurturin, Dlx5, and Ntrk2 performance. **(B)** Increased gadd45b expression in the brains of depressed mice after ect therapy boosts BDNF function by modifying BDNF demethylation. **(C)** Decreased Gadd45b, BDNF expression in the brain of patients with depression (Figure was created with BioRender.com).

Collectively, these findings indicate that Gadd45b could be a novel mediator of depression-like behaviors (Figure 3). Presently, the clinical effect of Gadd45b in the treatment of depression remains contentious and requires additional investigation in future trials.

### Autism

Autism is a neurodevelopmental condition characterized by language and social interaction deficits and is generally accompanied by seizures, cognitive deficits, or other brain disorders (Kidd, 2002). This condition is considered to be caused by hereditary factors and cerebral inflammation (Ratajczak, 2011). Some studies have revealed the abnormal DNA and histone methylation regulation in the prefrontal cortex of patients with autism (Nguyen et al., 2010; Shulha et al., 2012). Furthermore, A microarray analysis has detected elevated Gadd45b expression in the superior temporal gyrus of autism (Garbett et al., 2008).

**FIGURE 4:**
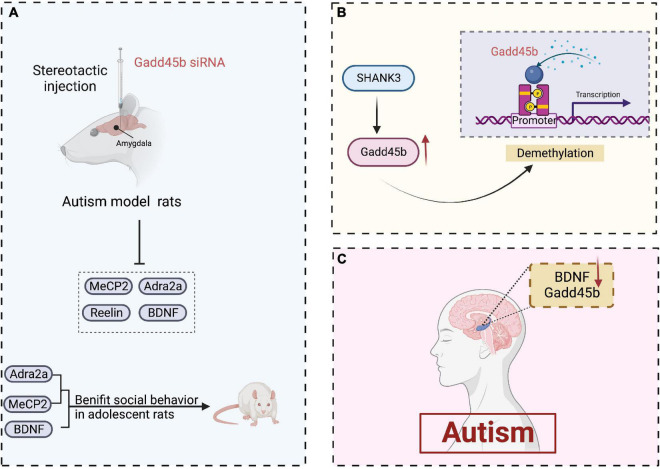
Roles of growth arrest and DNA damage-inducible beta (Gadd45b) in autism. **(A)** Stereotactic injection of gadd45b siRNA decreases the production of MeCP2, BDNF, Adra2a, and Reelin in autistic rats, where MeCP2, BDNF, and Adra2a contribute to adolescent rats’ social competence. **(B)** SHANK3 enhances gadd45b expression, which may be related to gadd45b demethylation. **(C)** Decreased Gadd45b and BDNF expression in the brain of patients with autism (Figure was created with BioRender.com).

Noteworthily, Gadd45b deletion in neonatal rats reduces the expression of numerous genes linked to psychiatric illnesses, including MeCP2, Reelin, and BDNF (Kigar et al., 2015). Similarly, Serum BDNF levels are decreased in patients with autism (Hashimoto et al., 2006; Abdallah et al., 2013). Also, Methyl-CpG-binding protein 2 (MeCP2) deletion in the developing amygdala limits social interaction in adolescent rats (Kurian et al., 2008). A2-adrenoceptor (Adra2a) is required to regulate social behavior in adolescent rats. Furthermore, Gadd45b siRNA therapy considerably reduced Adra2a expression in the juvenile rat amygdala (Siviy and Baliko, 2000). In addition, multiple ankyrin repeat domains protein 3 (SHANK3) is a potential autism-related gene. And it has been shown to be involved in the action of Gadd45b on DNA demethylation (Monteiro and Feng, 2017). Collectively, these findings suggest that Gadd45b has a vital role in the epigenetic process of autism (Figure 4). Still, its precise mechanism must be proven by conducting a large number of studies.

## The role of Gadd45b in neurologic disorders

### Stroke

Stroke is the leading cause of permanent severe disability. More than half of older than 65-year-old stroke survivors have reduced mobility. Approximately 87% of all strokes are ischemic stroke. Due to the time limits of surgical therapy and the danger of recombinant tissue-type plasminogen activator therapy, neuroprotectants that inhibit the inflammatory response to combat ischemic injury have recently emerged as a new direction in stroke treatment (Paul and Candelario-Jalil, 2021). In the past several decades, Gadd45b has been demonstrated as an inherent neuroprotective factor protecting retinal ganglion cells from injury (Liu et al., 2009, 2013). Therefore, Gadd45b may be a new target for stroke treatment.

Researchers reported the continuous detection of Gadd45 immunoreactivity in neurons within the infarct from 4 to 24 h and in sublethally injured neurons within the penumbra from 24 to 48 h (Charriaut-Marlangue et al., 1999). From this investigation, we reached the conclusion that the expression of the Gadd45 family changes after cerebral ischemia. Specifically, the term of the Gadd45 mRNA in the ischemic hemisphere increases 3 h after reperfusion and approximately three-fold after 24 h compared to the contralateral or sham-operated group (Hou et al., 1997). In addition, Jin et al. (1996) discovered that Gadd45 protein is continuously expressed only in the ischemic penumbra from 4 to 24 h after cerebral ischemia. Meanwhile, the evidence revealed that Gadd45 protein could promote injury repair and expressed at higher levels in surviving neurons after cerebral ischemia. Consequently, the Gadd45 protein may protect against neuronal injury caused by cerebral ischemia.

In addition, several newly published preclinical studies demonstrate that Gadd45b protects against cerebral ischemia damage in both transitory localized and global models of cerebral ischemia. Chen et al. (1998) discovered meaningful increases in the expression of the Gadd45 mRNA and protein in the brain after transient global cerebral ischemia. Liu and coworkers showed that cerebellar fastigial nucleus stimulation induces Gadd45b expression in the cerebral cortex after cerebral ischemia. Over-activation of Gadd45b promotes functional recovery after focal cerebral ischemia, implying that Gadd45b may be related to motor function after cerebral ischemia (Liu et al., 2012).

The recovery process after a stroke involves neurogenesis and axonal remodeling within the ischemic brain. Neurogenesis occurs following ischemic brain injury. Stroke generates a considerable increase in cell proliferation in the lateral ventricle’s subventricular zone (SVZ) in the ischemic penumbra. However, the intrinsic neurogenesis abilities are generally insufficient to improve functional recovery (Arvidsson et al., 2002). A recent study showed that Gadd45b is related to SVZ neurogenesis following middle cerebral artery occlusion (MCAO), and Gadd45b mediates neurogenesis in the SVZ *via* BDNF (Tan et al., 2021). Gadd45b has been shown to alter the methylation of BDNF following cerebral ischemia-reperfusion injury (He et al., 2015). Both activin receptor-like kinase 5 (ALK5) signaling and DNA demethylation are crucial in adult neurogenesis. ALK5 regulates the protein level and demethylation of BDNF through controlling the production of Gadd45b, consequently encouraging the recovery of brain function (Zhang et al., 2019). Axonal remodeling is also induced by cerebral ischemia. Liu et al. (2015a) discovered that Gadd45b leads to axonal regeneration and promotes functional recovery following MCAO in rats. Gadd45b regulated axon regeneration by BDNF and its downstream cAMP/PKA/CREB pathway after transient cerebral ischemia. In summary, Gadd45b could play a role in promoting axonal plasticity and neurogenesis. This procedure may enhance stroke recovery, which is important for brain recovery.

Neuronal apoptosis in ischemic brain penumbra is one of the major factors of neurological diseases. To investigate the role of Gadd45b in cerebral ischemia, lentiviral vector-associated RNA interference was injected stereotaxically into the ipsilateral lateral ventricle to inhibit Gadd45b expression. In this study, Gadd45b was shown to protect against rat brain ischemia injury by inhibiting apoptosis. The investigation suggested Gadd45b as a neuroprotective effector against apoptosis by regulating Bcl-2 and Bax (Liu et al., 2015b). In addition, Gadd45b expression is dramatically increased in hippocampal CA1 neurons from rats with transient global cerebral ischemia. Gadd45b protects neurons from ischemic injury by activating the DNA demethylation of the BDNF regulatory region in global ischemia-induced neuronal death (Cho et al., 2019). Equally, in an *in vitro* model of hypoxia and ischemia, Gadd45b was also shown to inhibit autophagy and alleviate neuron cell apoptosis (He et al., 2016).

**FIGURE 5:**
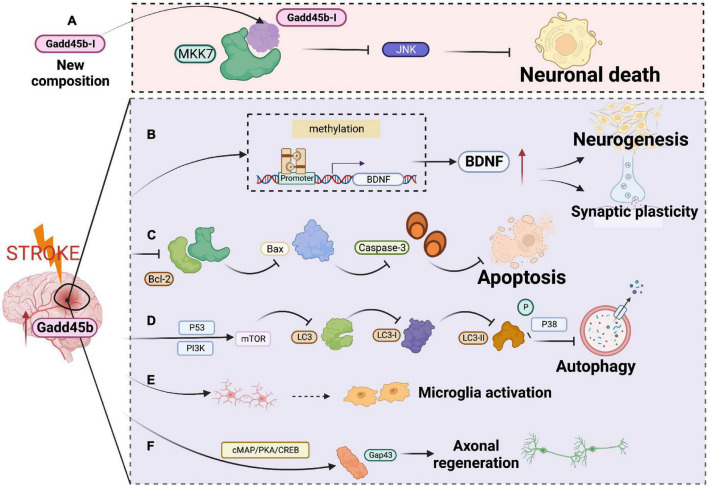
Roles of growth arrest and DNA damage-inducible beta (Gadd45b) in cerebral ischemic disease. **(A)** Gadd45b-I specifically binds to MKK-7 and inhibits JNK-mediated neural death. **(B)** Gadd45b promotes neurogenesis and synaptic remodeling by altering the demethylation of BDNF. **(C)** Gadd45b decreases the release of Bcl-2 and Bax to inhibit apoptosis. **(D)** Gadd45b inhibits cellular autophagy by stimulating PI3K/P53 signaling pathway and suppressing the P38 signaling pathway. **(E)** Gadd45b may be associated with microglia polarization. **(F)** Gadd45b promotes axonal regeneration through the camp/PKA/CREB signaling pathway (Figure was created with BioRender.com).

Of note, microglia activation is critical for attenuating neuronal apoptosis, enhancing neurogenesis, and facilitating functional recovery after cerebral ischemia. In a mouse model of transient focal cerebral ischemia, Guo et al. used unbiased single-cell sequencing in conjunction with bulk RNA-seq analysis to study microglia diversity at an early stage of ischemic stroke. They discovered that Gadd45b is overexpressed in microglia cells in the ischemic brain (Guo et al., 2021). Mitogen-activated protein kinase kinase-7 (MKK-7) is an upstream activator of the c-Jun-N-terminal kinase (JNK) signaling pathway, which is related to brain damage after acute cerebral ischemia. Fortunately, researchers have discovered Gadd45b-I, an effector peptide based on the Gadd45b domain (Vercelli et al., 2015). Gadd45-I was capable of binding to MKK7 and, by coupling it to the TAT peptide sequence, enabling membrane penetration. Studies *in vitro* reveal that Gadd45-I significantly lowers neuronal mortality generated by *N*-methyl-D-aspartate exposure or oxygen-glucose deprivation-induced excitotoxicity. In addition, Gadd45-I exhibited long-lasting neuroprotection against ischemia *in vivo*. Taken together, Gadd45b is a new putative target for ischemic stroke (Figure 5 and Table 1).

**TABLE 1:** Studies of growth arrest and DNA damage-inducible beta (Gadd45b) in cerebral ischemia.

Study type	Study (year)/references	Study findings
Rat global cerebral ischemia	Cho et al. (2019)	(1) Ischemic insults increase the abundance of Gadd45b and BDNF
		(2) Gadd45b blocks caspase activation and neuronal death
Rat tMCAO	Liu et al. (2012)	The expression of Gadd45b increased after transient focal cerebral ischemic injury
	Tan et al. (2021)	(1) Gadd45b increases neurogenesis in subventricular zone
		(2) Treatment with EE following ischemic stroke increases Gadd45b expression and neurogenesis in subventricular zone
		(3) Inhibition of Gadd45b ameliorates the increased neurogenesis induced by EE
	Zhang et al. (2019)	(1) ALK5 mediates Gadd45b protein levels by regulating Smad2/3 phosphorylation
		(2) ALK5 mediates neurogenesis and functional recovery *via* Gadd45b
	Liu et al. (2015)	(1) Gadd45b promotes axonal plasticity after MCAO
		(2) Gadd45b mediates axonal regeneration through BDNF and cAMP/PKA/pCREB pathway
	Liu et al. (2015) and Vercelli et al. (2015)	(1) Gadd45b prevents apoptosis
		(2) Gadd45b affects the expression of Bax and Bcl-2
		Gadd45b-I inhibits cell death by binding specifically to MKK7
OGD	He et al. (2016)	Gadd45b prevents autophagy and apoptosis
	He et al. (2015)	Gadd45b increases the BDNF and methylation level of the forth CpG islands

tMCAO, transient middle cerebral artery occlusion; EE, environmental enrichment; OGD, oxygen-glucose deprivation; BDNF, brain-derived neurotrophic factor; cAMP, cyclic adenosine monophosphate; PKA, protein kinase A; CREB, cAMP-responsive element binding protein.

### Seizure

A seizure is an abrupt, uncontrolled electrical disruption in the brain. Seizures are associated with several alterations in the neuroplasticity of hippocampal circuits, including neurogenesis and dendritic development in the dentate gyrus (Naegele, 2009). Seizures are among the most potent stimulators of neurogenesis, hastening the differentiation of adult-born neurons (Tongiorgi et al., 2004; McNamara et al., 2006). Similarly, electroconvulsive therapy-induced epileptic episodes have increased dentate granulosa cell neurogenesis (Madsen et al., 2000; Scott et al., 2000). Coincidentally, recent research has linked neurogenesis to Gadd45b (Ma et al., 2009). Seizures have also been shown to stimulate the transcription of the Gadd45 gene (Henshall et al., 1999).

It is well-established that matrix metallopeptidase 9 (MMP-9) is a critical protein inducing the development of epilepsy in humans and rodents, and excess MMP-9 enhances the incidence of seizures (Wilczynski et al., 2008; Konopka et al., 2013). The increased MMP-9 expression in the hippocampus induced by epileptogenesis results from a complex epigenetic mechanism. Further research revealed that Gadd45b accumulation *in vivo* is one of the mechanisms of MMP-9 demethylation (Zybura-Broda et al., 2016). The study also implied that Gadd45b production induced by status epilepticus is mainly limited to postmitotic granule cells in the dentate gyrus.

In addition, Gadd45b-induced demethylation of BDNF has been hypothesized as a potential mechanism for the SE-induced enhancement of neurogenesis in the dentate gyrus (Xiao et al., 2020). The authors elegantly noted that following the elimination of Gadd45b, the degree of SE-induced demethylations of BDNF effectively was increased bout 5-fold. The mechanism of SE-induced BDNF demethylation by Gadd45b involves binding to the BDNF-specific Oct4 promoter, which drives region-specific demethylation through DNA nucleotide and base excision repair pathways, similar to the known role of Gadd45b in 5-hmC excision.

**FIGURE 6:**
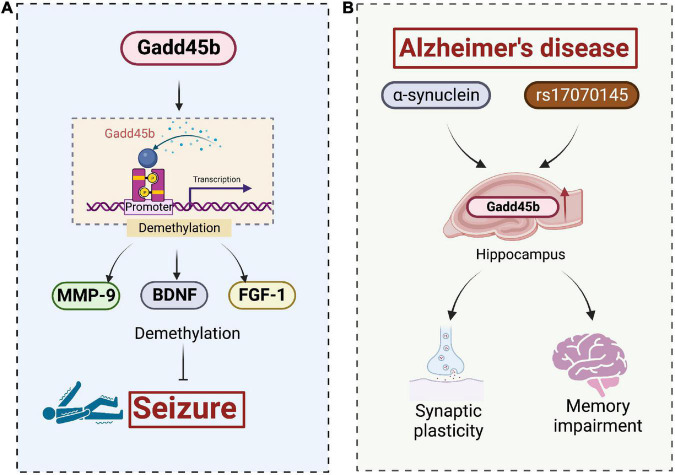
Roles of growth arrest and DNA damage-inducible beta (Gadd45b) in the seizures and Alzheimer’s disease. **(A)** Gadd45b suppresses seizures by altering the demethylation of MMP-9, BDNF, and FGF-1. **(B)** α-synuclein and rs17070145, recognized pathological features in AD, promote Gadd45b expression in hippocampal, facilitating synaptic remodeling and memory repairment (Figure was created with BioRender.com).

Ma et al. found that electroconvulsive treatment (ECT) in adult mice induced Gadd45b expression in granule cells of the hippocampal dentate gyrus with endogenous neural stem cell proliferation, new neuron formation, and temporal hippocampal dendrite growth. Gadd45b functions as an immediate-early gene by promoting endogenous gene (BDNF and FGF-1) demethylation. Knockdown of the Gadd45b gene in rats reduced ECT-induced proliferation of endogenous neural stem cells, neurite production, and dendritic growth in the hippocampus. The Gadd45b-induced increases in BDNF and FGF-1 expression are virtually eliminated in Gadd45b knockout rats. Based on these observations, Gadd45b could play a role in enhancing long-term neuroplasticity in the post-epileptic brain. This procedure, to some extent, facilitates structural repair of brain tissue and promotes functional recovery after epilepsy (Figure 6).

### Dementia

Dementia is a disorder defined by a deterioration in cognition relative to a prior level, impairing occupational, household, or social functioning (Gale et al., 2018). Memory loss is a crucial and early indication of dementia and occurs in conjunction with it.

Interestingly, a new modified porcine placenta extract improves age-related memory loss in mice by upregulating Gadd45b (Yamauchi et al., 2019). Meanwhile, Gadd45b knockout mice exhibit a hippocampus-dependent long-term memory impairment (Leach et al., 2012). Furthermore, Gadd45b deletion increases fear in a hippocampus-dependent manner and promotes memory and synaptic plasticity (Sultan et al., 2012). These studies implicate Gadd45b as a regulator of memory formation and may be a viable therapeutic target in cognitive disease.

The most widespread type of dementia is Alzheimer’s disease (AD). It is a progressive neurodegenerative disease of the brain that disrupts memory and other basic cognitive functions, leading to persistent mental decline and behavioral abnormalities that severely affect the daily life of patients. Deposing extracellular amyloid β-peptide (Aβ) plaques in the brain is a hallmark pathological feature of AD (Lambert et al., 1998). According to previous studies, Gadd45 is expressed in hippocampal CA1 pyramidal cells, which are susceptible to AD (Torp et al., 1998).

Alzheimer’s disease is characterized by the pathological combination of Aβ, tau, and α-synuclein. Mutations in the genes encoding Aβ, tau, and α-synuclein are common. Overexpression of these mutant proteins may result in a disease-associated phenotype (Goedert, 2015). Of note, extracellular α-synuclein induces the activation of Gadd45b (Motyl et al., 2018). The rs17070145-T variant of the WWC1 gene, which encodes the KIBRA protein, has a connection to improved episodic memory performance and a lower risk of late-onset AD, whereas rs17070145 increases Gadd45b expression (Piras et al., 2017). Thus, we infer that Gadd45b represents a potential target for AD treatment, but the exact mechanism remains to be investigated (Figure 6).

### Parkinson’s disease

**FIGURE 7:**
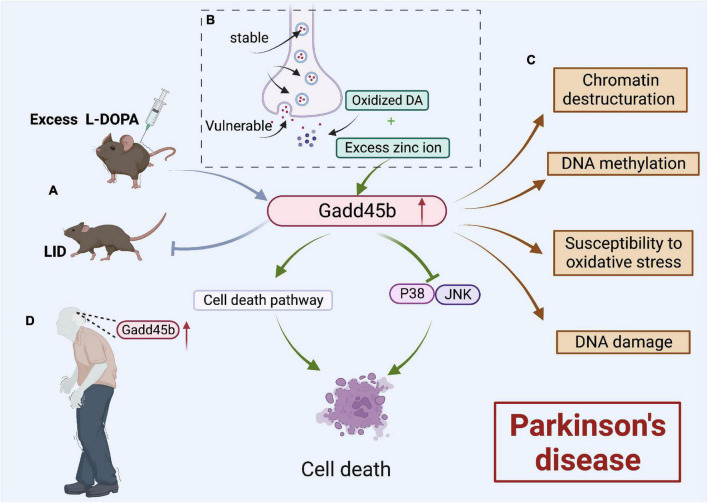
Roles of growth arrest and DNA damage-inducible beta (Gadd45b) in Parkinson’s disease. **(A)** Gadd45b could prevent LID caused by excessive L-DOPA. **(B)** Oxidized DA and Excess Zn2+ could increase Gadd45b, which induced cell death by inhibiting the JNK/P38 signaling pathway and promoting the cell death process. **(C)** Gadd45b may be related to chromatin destructuration, DNA methylation, susceptibility to oxidative stress and DNA damage. **(D)** Increased Gadd45b expression in the brain of patients with PD (Figure was created with BioRender.com).

Parkinson’s disease (PD) is a neurodegenerative disorder characterized by a significant loss of dopamine (DA) neurons in substantia nigra pars compacta (SNpc) (Benayoun et al., 2015). Studies showed that Gadd45b expression rose dramatically both in SNpc of patients with PD and mouse brains with age. Therefore, Gadd45b, a known DNA demethylation enzyme, may play a significant role in PD (Figure 7).

Recent research (Ravel-Godreuil et al., 2021) revealed that Gadd45b overexpression led to a variety of alterations in the striatum of PD model mice, including loss of Chromatin structure, altered DNA methylation, and increased vulnerability of mDA neurons to oxidative stress, and neuronal death. Clearly, they hypothesized that the Gadd45b activates methylation changes of LINE-1, a prospective DNA damage factor (Blaudin de Thé et al., 2018), altering the heterochromatic structure and causing neurological disease.

Dopamine is prone to oxidation when discharged into the cytoplasm or extracellular space. Both oxidized DA and excessive Zn2+ may contribute to neuronal death and cause Parkinson’s disease (Blum et al., 2001; Jenner, 2003). Yang indicated that Gadd45b could be the mediator of this route. It was found that the expression of Gadd45b was considerably boosted in PC12 cells induced by oxidized DA and excessive Zn2+. They hypothesized that Gadd45b induces PC12 death by blocking the P38/JNK signaling pathway and increasing the cell death process.

However, Gadd45b could be a potential target in countering dopamine precursor 3,4-dihydroxyphenyl-L-alanine (L-DOPA)-induced dyskinesia (LID) Parkinson’s disease (Park et al., 2016). Researchers developed a 6-OHDA animal model of hemi-parkinsonism. In the striatum of the model animals, prolonged overstimulation with L-DOPA raised Gadd45b mRNA levels through the R1D pathway (Heiman et al., 2014). Park et al. (2016) demonstrated that overexpression of Gadd45b inhibited the L-DOPA-induced elevation in c-Fos and FosB/FosB mRNA levels, which inhibited LID.

### Brain tumor

**FIGURE 8:**
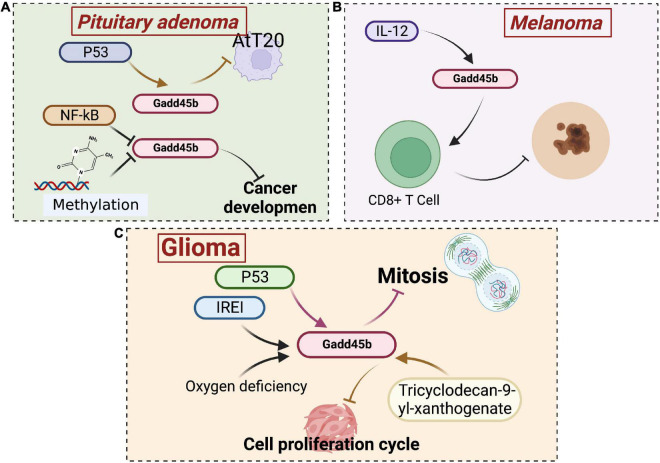
Roles of growth arrest and DNA damage-inducible beta (Gadd45b) in brain tumor. **(A)** Gadd45b is associated with NF-kB and P53 signaling pathways inhibiting pituitary adenoma cell growth. **(B)** The inhibitory effect of Gadd45b on melanoma growth is associated with IL-12. **(C)** The inhibitory effect of Gadd45b on glioma growth may be related to P53, IREI, and Tricyclodecan-9-yl-xanthogenate (Figure was created with BioRender.com).

Gadd45 is associated with the development of several oncological diseases. Numerous studies have shown that the Gadd45 protein is aberrantly expressed in pancreatic cancer, hepatocellular carcinoma, lung cancer, gastrointestinal tumors, and lymphoma. Upregulation of Gadd45 induces tumor cell cycle arrest and apoptosis. Coincidentally, the anticancer activity of chemotherapeutic agents (Rishi et al., 1999) and non-steroidal anti-inflammatory drugs depends on this effect (Zerbini et al., 2006). Therefore, Gadd45 has been identified as a potential target for antitumor drug therapy. The Gadd45b protein has been studied in neurological tumors, mainly in pituitary tumors, fibrillary astrocytoma, and adult neural tube cell tumors (Figure 8).

Human pituitary adenoma is a common intracranial tumor caused by hormone-secreting cells in the anterior pituitary (Ezzat et al., 2004). According to microarray studies, Gadd45b was downregulated 68-fold in pituitary tumors, and its loss may contribute to tumorigenesis or progression (Michaelis et al., 2011). Thus, this study identified Gadd45b as a novel pituitary tumor suppressor whose re-expression blocks proliferation, survival, and tumorigenesis. In addition, Gadd45b is a putative downstream target of protein 53 (p53) (Michaelis et al., 2011). Daptomycin has been shown to inhibit cell proliferation in AtT 20 corticotropic tumor cells *via* the p53-Gadd45b pathway, exerting anticancer effects (Kageyama et al., 2015). Furthermore, inhibiting the Gadd45 gene family through promoter methylation or nuclear factor kappa-B (NF-κB) activation is a critical step in cancer development (Tamura et al., 2012). In addition, p53 regulates Gadd45b expression through the NF-κB pathway (Zhai et al., 2019). Conversely, Gadd45b protein and mRNA expression are significantly reduced in human patients with gonadotrophic tumors. Gadd45b promotes gonadotrophic tumor apoptosis, primarily by arresting cells in G1/S and G2/M phases, but its upstream regulatory pathways are unknown (Zhang et al., 2002).

Gliomas are the most common primary tumors of the brain and spinal cord. The primary gliomas include astrocytic, oligodendrocyte, ependymal, neuronal, and mixed glial tumors (such as ganglioglioma) (Chen et al., 2017). Inhibition of the inositol requiring enzyme-1 (IRE1) pathway has been shown to substantially slow glioma growth in previous investigations (Drogat et al., 2007; Auf et al., 2010). Hypoxia is also related to the development of gliomas, and both hypoxia and IRE1 inhibition have been shown to promote Gadd45b gene expression in U87 glioma cells (Minchenko et al., 2016). Galectin-1 is an effective regulator of glioblastoma cell growth. Reduced production of the proangiogenic molecule Galectin-1 abolishes the level of the Gadd45b mRNA (Le Mercier et al., 2008). Gourabine E retards mitosis in human glioblastoma cell tumor cells by upregulating the expression of Gadd45b, thereby inhibiting tumor growth (Cheng et al., 2019). In addition, Tricyclodecan-9-yl-xanthogenate also blocks cell cycle progression and inhibits tumor cell growth by decreasing Gadd45b expression in glioma stem cell-like cells (Kalluri et al., 2017).

Primary intracranial melanoma is sporadic, accounting for only 0.07% of all intracranial tumors (Arai et al., 2018). Hiring A (HA) inhibits melanin production. Coincidentally, the expression of Gadd45b is increased drastically in B16 melanoma cells treated with HA. The dendritic morphology observed after HA treatment is most likely attributed to increased expression of Gadd45b. In general, the melanocyte dendritic morphology is directly related to melanogenic activity (Villareal et al., 2012). Gadd45b specifically stimulates p38 activation, increasing interferon γ (IFN-γ) levels in CD8+ T cells. Gadd45b expression is upregulated by interleukin-12 (IL-12), which increases TCR-triggered activation of p38 MAP kinase and thus encodes a key signaling protein required for effective anti-melanoma immune responses (Ju et al., 2009).

### Autoimmune diseases of the nervous system

Autoimmune diseases are disorders of the autoimmune system that cause the body to attack its organs, cells, and tissues. Because the specific biological pathogenesis of autoimmune diseases is unknown, the condition cannot be cured but only managed. In particular, the potential importance of epigenetic phenomena in autoimmune diseases has been highlighted (Wang et al., 2015). Interestingly, *in vivo* and *in vitro* studies have shown that immunization increases Gadd45b expression (Wang et al., 2006). Experimental studies have indicated that some autoimmune diseases are closely related to Gadd45b. For example, the Gadd45b mRNA is expressed at lower levels in patients with rheumatoid arthritis (RA) than in healthy people (Li et al., 2019). In particular, increased Gadd45b expression or inhibition of the activity of JNK or its upstream regulator MKK-7 alleviates the clinical symptoms of patients with RA (Svensson et al., 2009).

Neurological autoimmune diseases are diseases in which autoimmune cells and molecules are the main pathogenic factors attacking the nervous system. Multiple sclerosis (MS) is an immune-mediated inflammatory demyelinating disease of the central nervous system driven by autoreactive Th cells. Experimental allergic encephalitis (EAE) is an animal model of induced demyelinating disease with major symptoms that are very similar to those of humans with MS. Gadd45b deletion exacerbates EAE (Liu et al., 2005; Lu, 2006). Gadd45b is important for initiating TH1 cell responses by mediating pathogen-associated molecular pattern (PAMP) signaling in innate immune cells, enhancing and prolonging TCR signaling in T cells, and prolonging IL-12/IL-18 signaling in TH1 cells, which are important for initiating the TH1 response. TH1 cells are important components of chronic inflammation and are involved in the pathogenesis of autoimmune diseases (Svensson et al., 2009).

Additionally, myasthenia gravis (MG) is an autoimmune disease caused by dysfunctional neuromuscular junction transmission, and Gadd45b expression is downregulated in patients with thymoma presenting with myasthenia gravis (Yu et al., 2019). Thus, Gadd45b is involved in the regulation of several neurological autoimmune diseases. However, the specific mechanisms and functions of Gadd45b in these diseases have not been thoroughly established due to the lack of sufficient evidence.

## Conclusion

In summary, Gadd45b is widely expressed in the central nervous system, plays a vital role in various nervous system diseases, and is a potential target for the treatment of various nervous system diseases. Gadd45b could enhance the long-term neuroplasticity of the brain and exert a neuroprotective effect. Consistent with their discovery, knockdown or overexpression of Gadd45b could cause a range of neurodevelopmental outcomes. For instance, the phenotypes of Gadd45b in mice include aberrant dendritic cell physiology, inappropriate production of IL-4, IL-6, and IFN-γ, as well as enhanced T cell proliferation (McMurry et al., 2016; Shefchek et al., 2020). Gadd45b knockdown or knockout inhibited the activity-driven proliferation of neural progenitors and the dendritic formation of newborn neurons (Kaufmann et al., 2011). Still, we are unaware of any observed human phenotypes associated with the Gadd45 family. In addition, no PWAS research linking Gadd45 to human disorders has been discovered by the authors. GWAS have effectively revealed risk loci for a multitude of neuropsychiatric disorders and traits, such as anorexia nervosa, major depressive disorder, schizophrenia, insomnia, and Alzheimer’s disease, among others (Brandes et al., 2020). Therefore, future research is required to connect genes and phenotypes *via* functional changes in proteins, revealing insights into the architecture of the discovery of novel disease-causing genes and pathways as well as the identification of new therapeutic targets and disease biomarkers.

Noteworthy, multiple intriguing studies have displayed Gadd45b’s contribution to the growing subject of cognitive neuro-epigenetics. The most notable distinction between these processes and those previously addressed is their operation in normal, senescent adult neurons, as opposed to those undergoing intentional cell death or growth (Sultan and Sawaya, 2022). Also, disruptions in DNA methylation patterns have been linked to the development of neurologic and neuropsychiatric illnesses (Shirvani-Farsani et al., 2021). Drug addiction is a chronic psychiatric disorder in which global and site-specific changes in DNA methylation can be observed (Wang et al., 2012). In addition, there is a substantial correlation between BDNF promoter methylation and the likelihood of drug abuse (Wang et al., 2012). However, only a single study has shown that striatal Gadd45b operates as a dopamine-induced gene that is required for cocaine reward memory through modulating DRD1 transcriptional activity (Zipperly et al., 2021). Therefore, the exploration of Gadd45 in its field holds great promise.

In addition, the breadth of loci targeted by Gadd45b for epigenetic regulation is largely unknown and a subject for future investigation. Fortunately, certain therapies, such as electroconvulsive treatment, have enhanced Gadd45b expression. Of note, researchers have discovered Gadd45b-I, an effector peptide based on the Gadd45b domain. Therefore, we conclude that using Gadd45b as a target for the therapy of neurologic and neuropsychiatric diseases may have far-reaching implications.

## Author contributions

X-YS and S-HS were responsible for literature review, investigation, and draft preparation. BL was responsible for literature review and editing. Y-BL was responsible for drawing and editing. All authors contributed to the article and approved the submitted version.

## Abbreviations

Gadd45b: growth arrest and DNA damage-inducible beta
BDNF: brain-derived neurotrophic factor
mGlu2: metabotropic glutamate receptor 2
GAD67: glutamate decarboxylase 67
TGF-β: transforming growth factor β
ECT: electroconvulsive therapy
Ntrk2: neurotrophic tyrosine kinase receptor type 2
Dlx5: distal-less homeobox 5
MeCP2: methyl-CpG-binding protein 2
Adra2a: A2-adrenoceptor
SVZ: subventricular zone
ALK5: activin receptor-like kinase 5
MMP-9: matrix metallopeptidase 9
p53: protein 53
NF-κB: nuclear factor kappa-B
HA: Hirsein A
IFN-γ: interferon γ
AD: Alzheimer’s disease
Aβ: amyloid β-protein
EAE: experimental allergic encephalitis
MG: myasthenia gravis.
